# Drug‐Induced Liver Injury Associated With Turmeric and Black Pepper Based Dietary Supplements Consumption: A Case Report

**DOI:** 10.1002/ccr3.71489

**Published:** 2025-11-16

**Authors:** Sébastien Pugnale, Léa Schilter, Ludovic Galofaro

**Affiliations:** ^1^ Department of Emergency Medicine University and Teaching Hospital Fribourg Switzerland; ^2^ Faculty of Science and Medicine University of Fribourg Fribourg Switzerland; ^3^ Department of Internal Medicine University and Teaching Hospital Fribourg Switzerland

**Keywords:** black pepper, curcumin, dietary supplements, drug‐induced liver injury (DILI), hepatotoxicity, herb‐induced liver injury (HILI), self‐medication, turmeric

## Abstract

For centuries, turmeric has been utilized for its therapeutic properties; however, it is now being more frequently associated with the development of drug‐induced liver injury (DILI). The popularity of dietary supplements (DS) is rising, yet patients frequently omit disclosing their use to healthcare providers, as they do not perceive them as drugs. This dynamic will expose doctors to more patients suffering from the adverse effects of DS consumption. We present the case of a 61‐year‐old white European male, presenting with right upper quadrant abdominal pain, initially managed with antibiotic therapy based on radiological evidence of acute cholecystitis. The clinical course was complicated by progressive jaundice and severe hepatitis, necessitating hospital admission for comprehensive diagnostic evaluation. Despite an exhaustive assessment, a definitive etiology was elusive until the patient revealed self‐administration of a DS comprising turmeric and black pepper over the preceding year as adjunctive treatment for depressive symptoms. This disclosure led to the diagnosis of DILI, and complete normalization of hepatic function was observed within 2 months following discontinuation of the DS. This case underscores the potential for turmeric‐containing DS, particularly those combined with bioavailability enhancers such as black pepper, to trigger DILI in patients at risk. Healthcare professionals should proactively inquire about their patients' DS consumption and remain vigilant for their potential adverse effects.

**Summary**

- Dietary supplements are increasingly taken by patients for health benefits, although they can have serious adverse effects.
- Doctors must be aware that curcumin may cause drug‐induced liver injury in patients at risk and must specifically ask their patients about their consumption of dietary supplements.

## 1 Introduction

Turmeric has been used for centuries for its culinary properties, as well as for the therapeutic properties of its bioactive compound, curcumin, in phytotherapy, including its anti‐allergic, antibacterial, anti‐inflammatory, antioxidant, and hypolipidemic effects [1, 2, 3]. Numerous studies have even revealed a hepatoprotective or even curative effect in certain cases of hepatotoxicity [4]. All these purported benefits probably contributed to the increase in turmeric sales worldwide, as well as its consumption as a dietary supplement (DS) [3, 5, 6], although there is currently no solid scientific evidence as to the pharmacological mechanisms explaining the effects described, nor any proof of efficacy [7]. It is only recently that the notion of drug‐induced liver injury (DILI) associated with turmeric consumption has emerged in the literature, with more and more cases being described [2, 8, 9] internationally. However, no cases have been reported in Switzerland to date.

As consumption of DS is increasing [3, 5, 6], healthcare professionals will be more frequently confronted with patients suffering from their adverse effects. Meanwhile DS are often self‐administered and omitted by patients when asked about their medication, mainly due to a lack of knowledge and consideration for their potential toxicity by the patient and a lack of specific questioning by the doctors [10]. Furthermore, DS are not subject to the same strict rules concerning pre‐marketing studies and the monitoring of adverse effects [7, 8]. All the above makes the diagnosis of DILI linked to DS consumption even more challenging and emphasizes the need for more scientific evidence on the subject.

This is the first case report in Switzerland of a DILI linked to the self‐administration of DS containing turmeric and black pepper. We aim through it to raise awareness about the potential hepatotoxicity of turmeric‐based DS in patients at risk, especially when combined with bioavailability enhancers such as black pepper, and to highlight the crucial need for healthcare professionals to specifically ask their patients about their self‐medication and DS use in order not to delay or miss this diagnosis.

## 2 Case History/Examination

A 61‐year‐old white European male, known for metabolic syndrome (BMI 29.3 kg/m^2^, hypertriglyceridemia, HDL‐hypercholesterolemia and systolic hypertension) and a high‐risk alcohol consumption (4–5 alcohol units per day), presented with progressive abdominal pain in the right hypochondrium for a week, prompting a consultation with his general practitioners, who retained the diagnosis of cholecystitis after an abdominal CT, for which co‐amoxicillin was prescribed. Because of persistent pain and the appearance of jaundice after 3 days of antibiotic therapy, he was referred to the emergency department. He described no fever, a slight clarification of the stool, and no melena, as well as dysuria, pollakiuria and dark urine for several days. The rest of the systematic anamnesis revealed a significant disabling fatigue and loss of appetite for 1 year, with a loss of 6 kg in 6 months. There was no recent change in his prescribed medication.

On clinical examination, the abdomen was painful in the right hypochondrium and right iliac fossa, without guarding or rebound tenderness, and a negative Murphy's sign. The cardiovascular status revealed slight oedema of the lower limbs. The rest of the clinical examination revealed no significant abnormalities.

The laboratory tests showed aspartate aminotransferase (AST) of 1350 U/L, alanine transaminase (ALT) of 2534 U/L, alkaline phosphatase (ALP) of 214 U/L, gamma‐glutamyl transferase (GGT) of 550 U/L, total bilirubin (TB) of 247 mcmol/L and direct bilirubin (DB) of 172 mcmol/L, with preserved synthetic liver function. The R factor for liver injury was 39.9, indicating a hepatocellular injury [11, 12].

An abdominal ultrasound confirmed signs of acute cholecystitis without evidence of gallstones, or other liver parenchyma or biliary duct abnormality motivating the pursuit of co‐amoxicillin. The renal and pancreatic function as well as the complete blood count were normal. We therefore retained the diagnosis of acute hepatitis.

## 3 Differential Diagnosis, Investigations and Treatment

In the initial diagnostic approach, a viral origin (HAV, HBV, HCV, HIV, CMV, EBV, HSV, VZV serologies were negative) as well as an autoimmune origin (the screening for autoimmune hepatitis, the antinuclear antibody and the screening for antineutrophil cytoplasmic antibody vasculitis were negative) were excluded. Ferritin was high (11′601 mcg/L) without argument for hemochromatosis. An alcoholic origin was less probable considering an AST/ALT ratio < 1 [13].

As the cholestasis parameters continued to deteriorate, a second abdominal ultrasound was performed on the third day but did not show new findings, specifically no signs of bile duct obstruction, hepatic steatosis or cirrhosis. The etiological work‐up was extended to exclude Coxiella Brunetti or HEV infection (negative serology and PCR respectively), Wilson's disease (ceruloplasmin in the normal range) and alpha‐1‐antitrypsin deficiency. As a further step, a magnetic resonance cholangiopancreatography was performed on the sixth day but detected no signs of bile duct obstruction and revealed no abnormalities in the liver parenchyma suggestive of steatosis or liver cirrhosis.

**FIGURE 1:**
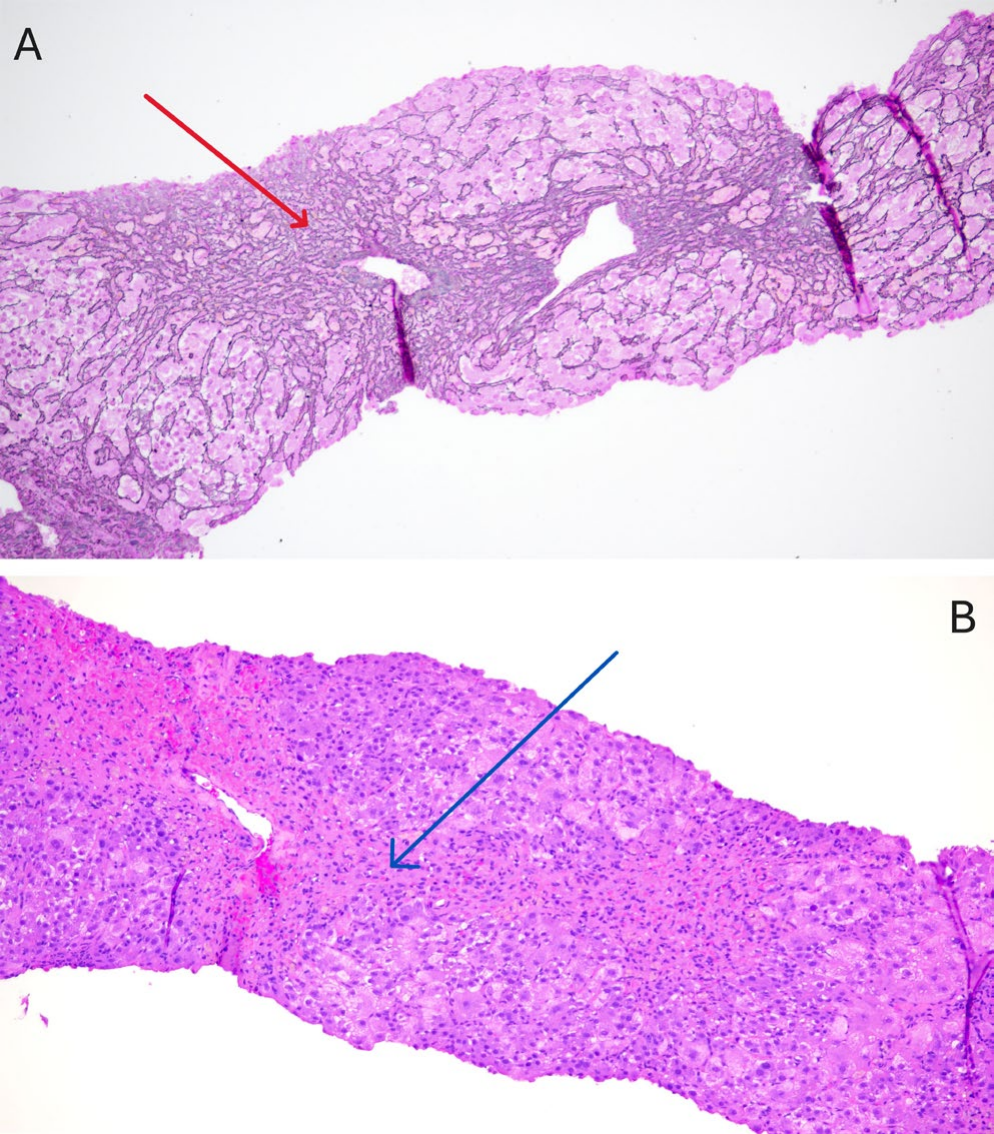
(A) Photomicrographs magnified 10× of reticulin core liver biopsies showing lobular hepatitis with areas of centrilobular parenchymatous collapse (red arrow). (B) Photomicrographs magnified 10× of hematoxylin and eosin‐stained core liver biopsies showing centrilobular necrosis in bridges (blue arrow). This histopathological presentation is suggestive of a drug‐induced origin [14].

Faced with a patient showing only minor improvement in cytolysis and a worsening of cholestasis in the absence of any identified etiology, a liver biopsy (Figure 1) was performed. It showed lobular hepatitis with areas of centrilobular parenchymatous collapse in bridges, testifying to subacute centrilobular necrosis, and clear bilirubin stasis, suggestive of a drug‐induced origin. In addition, the biopsy showed no signs of hepatic fibrosis, cirrhosis or steatosis.

## 4 Outcome and Follow‐Up

**FIGURE 2:**
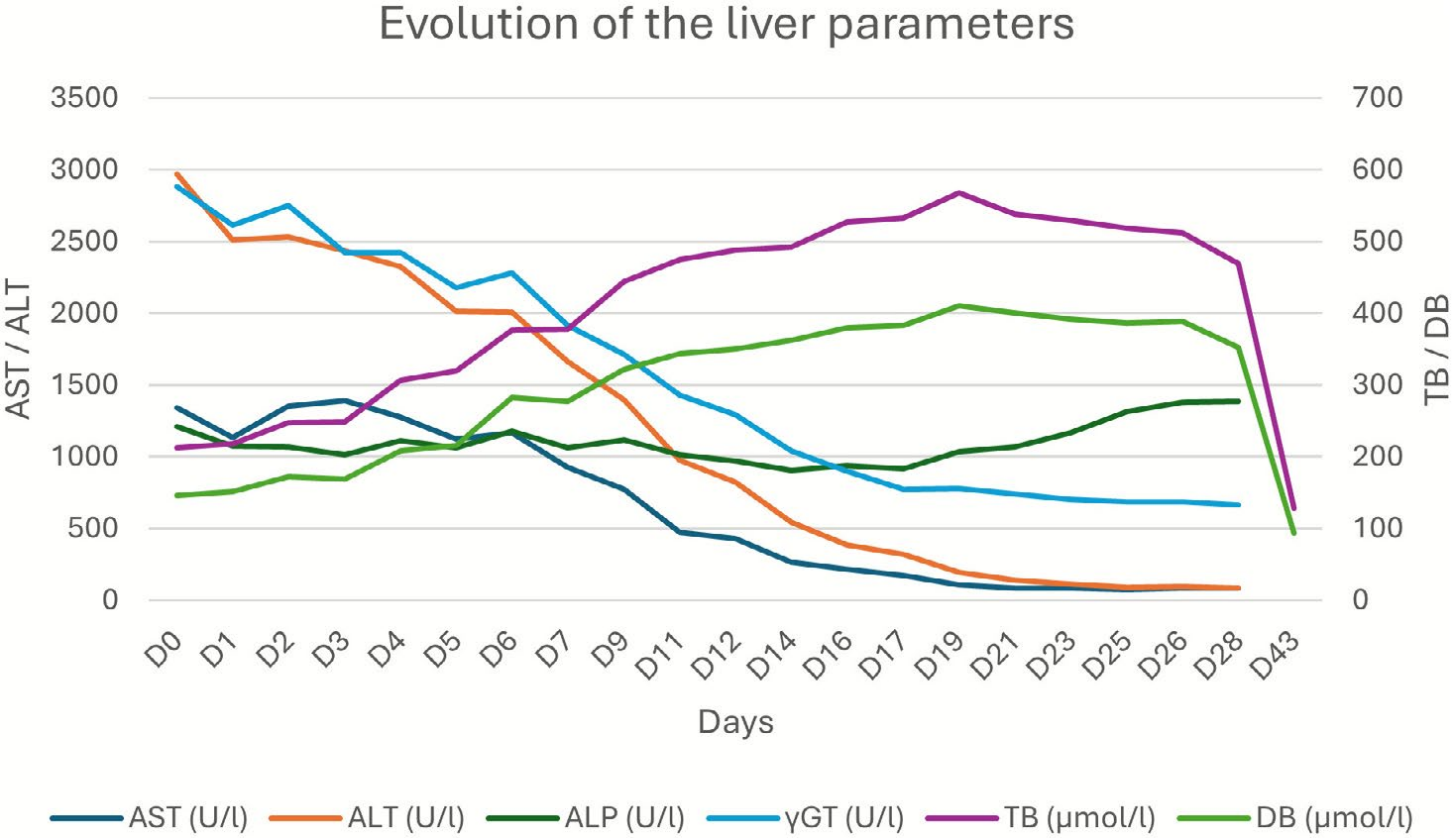
Graphical evolution of the liver parameters. D0 (Day 0) represents the day of admission. The patient was discharged on D28 with a first outpatient liver function check on D43. ALP, alkaline phosphatase; ALT, alanine transaminase; AST, aspartate transferase; DB, direct bilirubin; TB, total bilirubin; γGT, gamma‐glutamyl transferase.

After expanding on the anamnesis concerning DS, the patient mentioned that he had been taking them for a year, on the advice of a work colleague, as a form of self‐medication for his fatigue and low mood, as his general practitioner had not taken his complaints into consideration. He specified that he had been taking one pill a day of a DS containing curcuma and black pepper for a year (Per pill: 250 mg curcuma powder, 50 mg ginger powder, 20 mg black pepper powder, 5 mg zinc citrate, 40 mg vitamin C) whose consumption ceased just before the hospitalization. The liver parameters improved without needing any specific treatment (i.e., positive dechallenge) (Figure 2).

The Roussel Uclaf Causality Assessment Method (RUCAM) [15] score was 8 (Table 1), consistent with probable drug‐induced liver injury (DILI), including herb‐induced liver injury (HILI). The biological follow‐up at 2 weeks after discharge (D43) showed nearly normal liver parameters, in favor of our retained diagnosis (Figure 2).

**TABLE 1:** Roussel Uclaf Causality Assessment Method (RUCAM) for drug‐induced liver injury/herb‐induced liver injury (hepatocellular injury).

		Points	Case report
Timing from drug start	5–90 days (rechallenge 1–15 days)	2	
	< 5 or > 90 days (rechallenge > 15 days)	1	X
Timing from drug cessation	< 15 days	1	
Difference between peak ALT and ULN value after drug stop	Decrease > 50% in 8 days	3	
	Decrease > 50% in 30 days	2	X
	Decrease > 50% in > 30 days	1	
	Decrease < 50% in > 30 days	−2	
Risk factors	Alcohol use—presence	1	X
	Alcohol use—absence	0	
Age	> 50 years	1	X
	< 50 years	0	
Concomitant drug(s)/herb(s)	None or no information	0	
	Concomitant drug(s)/herb(s) with incompatible time to onset	0	X
	Concomitant drug(s)/herb(s) with compatible or suggestive time to onset	−1	
	Concomitant drug(s)/herb(s) known as hepatotoxin and with compatible or suggestive time to onset	−2	
	Concomitant drug(s)/herb(s) with evidence for its role in this case (positive rechallenge of validated test)	−3	
Alternative causes^a^	All causes reasonably ruled out (group I and II)	2	X
	All causes of group I ruled out	1	
	5–6 causes of group I ruled out	0	
	< 5 causes of group I ruled out	−2	
	Alternative cause highly probable	−3	
Previous hepatotoxicity of drug/herb	Reaction labeled in the product characteristics	2	
	Reaction published but unlabelled	1	X
	Reaction unknown	0	
Response to unintentional re‐exposure	Doubling of ALT with drug/herb alone (if ALT < 5 N before re‐exposure)	3	
	Doubling of ALT with drug/herb already given at time of first reaction	1	
	Increase of ALT but < 5 N in the same conditions as for first administration	−2	
	Other situations	0	X
Total^b^			8

Abbreviations: ALT, alanine transaminase; ULN, upper limit of the norm.

^a^ Group I: hepatitis A virus, hepatitis B virus, hepatitis C virus, biliary obstruction (imaging), alcoholism, and acute recent hypotension. Group II causes are complications of underlying disease such as sepsis, cytomegalovirus, Epstein–Barr virus, and herpes simplex virus.

^b^ ≤ 0, excluded causality; 1–2, unlikely; 3–5, possible; 6–8, probable; ≥ 9, highly probable.

## 5 Discussion

DS are increasingly criticized for the side effects they can cause, up to and including severe damage [2]. As DS are not recognized as drugs, they are not subject to the same strict rules governing design, marketing and monitoring of adverse events (AE), resulting in dosage variability between different formulations without clear scientific evidence and under‐reporting of AE after marketing [7, 8]. This lack of regulation, coupled with the worldwide increase in consumption, is likely to lead to a rise in HILI cases, reinforcing the need to identify patients at risk.

In the case of turmeric, a consumption of up to 6 g of curcumin per day for 4–7 weeks is considered safe [2]. However, its bioavailability varies greatly depending on the components with which it is combined [1, 16]. Noteworthy, when combined with black pepper, as in the case described above, the bioavailability of curcumin increases by up to 2000%, with a consequent higher risk of HILI [16].

The literature is currently rather sparse on the pathophysiological mechanisms of hepatotoxicity in the case of turmeric‐induced liver injury [7], making it harder to identify risk factors. Several studies have shown an association with the presence of HLA B35:01, suggesting an immuno‐mediated mechanism. It is hypothesized that by interacting with the HLA, turmeric or its components induces T‐cell activation with hepatic autoantigen recognition leading to liver damage [7]. In the case of DILI, advanced age, female gender and alcohol consumption have been described as drug‐specific risk factors [12], and we can speculate that these may be extended to HILI, although future studies are needed to support this. From a histopathological perspective, a recent series of cases has shown that turmeric hepatotoxicity most often presents as pan‐lobular inflammation or inflammation predominantly in zone 3, as well as bridging centrilobular necrosis [14].

We report here the case of a 61‐year‐old man who presented with acute hepatitis which, after numerous investigations, possibly had a multifactorial origin. Although the patient did not meet the criteria for MASLD [17], as the various imaging tests and biopsy showed no signs of hepatic steatosis or cirrhosis, his metabolic syndrome and alcohol consumption, both known risk factors, potentially increased the risk of DILI in connection with the exposure to the turmeric‐based DS [12]. Nevertheless, in view of an AST/ALT ratio of < 1 [13] making an alcoholic etiology less likely, the RUCAM score of 8 suggestive of a probable causality with the exposure to the DS [15], the spontaneous improvement in liver function upon cessation of the DS (positive dechallenge) and the liver biopsy showing signs of DILI with an injury pattern consistent with turmeric hepatotoxicity [14], without signs of liver cirrhosis, we have retained the diagnosis of DILI, whose primary etiology was the consumption of the DS containing turmeric and black pepper.

The patient initially failed to mention self‐medication with DS, as he did not consider it to be a drug, did not believe it could be dangerous, and had not been specifically asked about it by the medical team. Furthermore, according to him, self‐medication stemmed from a lack of consideration on the part of the healthcare professionals he consulted regarding his fatigue and depressive symptoms. As a result, the diagnosis was probably delayed and required numerous aetological investigations.

All this highlights, on one hand, the need to raise awareness among healthcare professionals about the potential adverse effects of turmeric, especially when combined with bioavailability enhancers such as black pepper in DS, and on the other hand the need for systematic and specific questioning about the consumption of DS, especially when confronted with patients at risk [12, 18].

## Author Contributions

**Sébastien Pugnale:** conceptualization, data curation, formal analysis, investigation, methodology, project administration, resources, validation, visualization, writing – original draft, writing – review and editing. **Léa Schilter:** conceptualization, methodology, project administration, supervision, validation, writing – review and editing. **Ludovic Galofaro:** conceptualization, funding acquisition, methodology, project administration, supervision, validation, writing – review and editing.

## Ethics Statement

Ethics committee approval was not required for this case report in accordance with the institutional policies, as it presents a single anonymized patient case with informed consent.

## Consent

Patient consent to publish clinical information and images was obtained in written form using a consent form in French. The patient has agreed to the terms outlined in Wiley's standard consent form, including understanding that their medical information will be published on an open access basis and may be freely accessed worldwide.

## Conflicts of Interest

The authors declare no conflicts of interest.

## Data Availability

Data sharing is not applicable to this article as no datasets were generated or analyzed during the current study.
